# Deregulation of Mitochondria-Shaping Proteins Opa-1 and Drp-1 in Manganese-Induced Apoptosis

**DOI:** 10.1371/journal.pone.0091848

**Published:** 2014-03-14

**Authors:** Agustina Alaimo, Roxana M. Gorojod, Juan Beauquis, Manuel J. Muñoz, Flavia Saravia, Mónica L. Kotler

**Affiliations:** 1 Laboratorio de Apoptosis en el Sistema Nervioso y Nano-Oncología, Departamento de Química Biológica, Facultad de Ciencias Exactas y Naturales, Universidad de Buenos Aires and Instituto de Química Biológica, Ciencias Exactas y Naturales (IQUIBICEN-CONICET), Buenos Aires, Argentina; 2 Laboratorio de Neurobiología, Departamento de Química Biológica, Facultad de Ciencias Exactas y Naturales, Universidad de Buenos Aires and Instituto de Biología y Medicina Experimental (IBYME-CONICET), Buenos Aires, Argentina; 3 Departamento de Fisiología, Biología Molecular y Celular and Instituto de Fisiología, Biología Molecular y Neurociencias (IFIBYNE-CONICET), Facultad de Ciencias Exactas y Naturales, Universidad de Buenos Aires, Buenos Aires, Argentina; National Institute of Environmental Health Sciences, United States of America

## Abstract

Mitochondria are dynamic organelles that undergo fusion and fission processes. These events are regulated by mitochondria-shaping proteins. Changes in the expression and/or localization of these proteins lead to a mitochondrial dynamics impairment and may promote apoptosis. Increasing evidence correlates the mitochondrial dynamics disruption with the occurrence of neurodegenerative diseases. Therefore, we focused on this topic in Manganese (Mn)-induced Parkinsonism, a disorder associated with Mn accumulation preferentially in the basal ganglia where mitochondria from astrocytes represent an early target. Using MitoTracker Red staining we observed increased mitochondrial network fission in Mn-exposed rat astrocytoma C6 cells. Moreover, Mn induced a marked decrease in fusion protein Opa-1 levels as well as a dramatic increase in the expression of fission protein Drp-1. Additionally, Mn provoked a significant release of high MW Opa-1 isoforms from the mitochondria to the cytosol as well as an increased Drp-1 translocation to the mitochondria. Both Mdivi-1, a pharmacological Drp-1 inhibitor, and rat Drp-1 siRNA reduced the number of apoptotic nuclei, preserved the mitochondrial network integrity and prevented cell death. CsA, an MPTP opening inhibitor, prevented mitochondrial Δψm disruption, Opa-1 processing and Drp-1 translocation to the mitochondria therefore protecting Mn-exposed cells from mitochondrial disruption and apoptosis. The histological analysis and Hoechst 33258 staining of brain sections of Mn-injected rats in the striatum showed a decrease in cellular mass paralleled with an increase in the occurrence of apoptotic nuclei. Opa-1 and Drp-1 expression levels were also changed by Mn-treatment. Our results demonstrate for the first time that abnormal mitochondrial dynamics is implicated in both *in vitro* and *in vivo* Mn toxicity. In addition we show that the imbalance in fusion/fission equilibrium might be involved in Mn-induced apoptosis. This knowledge may provide new therapeutic tools for the treatment of Manganism and other neurodegenerative diseases.

## Introduction

Although manganese (Mn) is an essential metal required for diverse biological processes, it is also a common environmental pollutant. Chronic occupational exposure to high levels of Mn may cause its accumulation in the central nervous system (CNS), predominantly in the basal ganglia, resulting in Manganism or Mn-induced Parkinsonism [1], [2]. Clinical manifestations of Mn poisoning resemble the signs and symptoms of idiopathic Parkinson’s Disease (PD) suggesting a similarity between both diseases injury mechanisms. However, these neurodegenerative processes can be distinguished by analyzing basal ganglia damaged structures [2], [3]. While PD is characterized by the degeneration of dopaminergic neurons in the substantia nigra, several studies have indicated that GABAergic neurons from globus pallidus and striatum are earlier and more sensitive Mn targets [2], [4]. In addition, it has been suggested that dopaminergic neurons could also be damaged by Mn *in vivo*, an effect mediated by microglial cells and their associated activation products [5].

Although Mn may be incorporated into neurons and astrocytes, the latter represent the major storage site for this metal in the brain [6], [7]. Most of the Mn uptaked by astrocytes is sequestered by mitochondria and lysosomes while the rest remains in the cytosol [8], [9]. Mitochondria are morphologically dynamic organelles that continuously undergo two opposing events (fission and fusion) that operate in equilibrium to form small individual units or interconnected networks [10]. These processes play critical roles in mitochondrial functions and are controlled by GTPases, an evolutionary conserved large family of proteins. In mammals, optic atrophy-1 (Opa-1), mitofusin-1 (Mfn-1) and mitofusin-2 (Mfn-2) promote fusion whereas dynamin-related protein-1 (Drp-1) participates in mitochondrial fission by interacting with fission related protein-1 (Fis-1) and mitochondrial fission factor (MFF) [11], [12].

During apoptosis, mitochondria undergo prominent structural changes including fragmentation and cristae remodelling that ultimately mobilize the major pool of cytochrome *c* to the cytosol [13]. Whether or not the mitochondria-shaping proteins modulate the apoptotic mitochondrial pathway events still remains a subject of intense debate [11]. Several studies have implicated aberrant mitochondrial dynamics with exacerbated fission in the pathogenesis of neurodegenerative diseases such as Autosomal Dominant Optic Atrophy (ADOA), PD, Alzheimer’s Disease (AD), Huntington’s Disease and Charcot-Marie-Tooth [14], [15]. Consequently, mitochondrial dynamics is considered as a new paradigm for the research of neurodegenerative diseases.

It is well known that Mn exerts its effects, at least, by inducing mitochondrial dysfunction which includes mitochondrial respiration chain disruption, opening of mitochondrial permeability transition pore (MPTP) and loss of mitochondrial membrane potential (Δψm). All these events result in oxidative stress and the subsequent induction of signal transduction pathways that trigger apoptosis [16]–[19]. Recently, we reported the occurrence of mitochondrial fragmentation during Mn-induced apoptosis in C6 cells [19]. The present study was designed to investigate whether Opa-1 and/or Drp-1 expression levels are de-regulated in Mn-induced apoptosis employing both, *in vitro* and *in vivo* models. In addition, we studied the effect of these mitochondria-shaping proteins on *in vitro* apoptosis induced by Mn.

In this report we demonstrated for the first time that mitochondrial morphology alterations observed in Mn-induced apoptosis are paralleled by the Opa-1 and Drp-1 deregulation. These results would have relevant implications for the design of new therapeutic strategies in the treatment of Manganism and other neurodegenerative diseases in which the mitochondrial dynamics imbalance is involved.

## Materials and Methods

### Ethics Statement

Two month-old male Sprague-Dawley rats (n = 8) were obtained from the IBYME Animal Facility (NIH Assurance Certificate # A5072-01) and were housed under controlled conditions of temperature (22°C) and humidity (50%) with 12 hours/12 hours light/dark cycles (lights on at 7∶00 am). Prior to euthanization, rats were deeply anesthetized with a Xylazine (20 mg/kg)/Ketamine (50 mg/kg) cocktail by intravenous injection. Treated animals were perfused with 4% paraformaldehyde and brains were dissected. All *in vivo* experiments were performed according to the NIH Guide for the Care and Use of Laboratory Animals and were approved by the Ethical Committee of the Institute of Biology and Experimental Medicine (IBYME-CONICET, Argentina). All efforts were made to minimize animal suffering and to reduce the number of rats employed.

### Reagents

Dulbecco’s Modified Eagle’s Medium (DMEM), manganese chloride, 3-(4,5-dimethyl-thiazol-2-yl)-2,5-diphenyl-tetrazolium bromide (MTT), Hoechst 33258 fluorochrome, ECL detection reagents (luminol and p-coumaric acid) and 3,3′-diaminobenzidine (DAB) were purchased from Sigma Chemical Co. (St. Louis, MO, USA). Opti-MEM and Lipofectamine 2000 transfection reagent were from Life Technologies (Carlsbad, CA, USA). Fetal bovine serum (FBS) was obtained from BIO-NOS (Buenos Aires, Argentina) and horse serum was Gibco (26050-088). N-(2-hydroxyethyl) piperazine-N’-(2-ethanesulfonic acid) (HEPES) was from ICN Biomedicals (Irvine, CA, USA), 3-(2,4-Dichloro-5-methoxyphenyl)-2,3-dihydro-2-thioxo-4(1H)-quinazolinone (Mdivi-1) was purchased from Santa Cruz Biotechnology Inc. (Santa Cruz, CA, USA), rat Drp-1 siRNA (DNM1L) SiGENOME SMART pool was from Dharmacon, Thermo Scientific (Lafayette, CO. USA) and Cyclosporine A was obtained from Sandimmun Novartis Pharmaceuticals (Buenos Aires, Argentina). The mitochondria-specific red fluorescent probe MitoTracker Red CMXRos was from Molecular Probes (Eugene, OR, USA). ABC kit was from Vector Laboratories (Burlingame, CA, USA). Mdivi-1 was dissolved in dimethyl sulfoxide (DMSO) at a final concentration <0.25%, that did not affect cell viability and morphology. Drp-1 siRNA was dissolved in RNase-free water (pH 7.5) at a final concentration of 20 µM (stock) according to manufacturer’s protocol. All others chemicals used were of the highest purity commercially available.

### Cell Culture and Treatments

Rat astrocytoma C6 cell line (ATCC-CCL-107), originally derived from an N-nitrosomethylurea-induced rat brain tumor [20] was kindly provided by Dr. Zvi Vogel (Weizmann Institute of Science, Rehovot, Israel). C6 cells were maintained in DMEM supplemented with 10% heat-inactivated FBS, 2.0 mM glutamine, 100 units/ml penicillin, 100 µg/ml streptomycin and 2.5 µg/ml amphotericin B. Cells were cultured at 37°C in a humidified atmosphere of 5% CO_2_- 95% air, and the medium was renewed three times a week. For all experiments, C6 cells were removed with 0.25% trypsin, diluted with DMEM 10% FBS and re-plated into 12- well plates (1.2×10^5^ cells/well) or 96- well plates (2×10^4^cells/well) to yield 70–80% confluent cultures after 24 hours. Then, cells were washed with phosphate buffered saline (PBS) and then exposed to 750 µM MnCl_2_ for 24 hours in serum free medium.

### Plasmids and Transient Transfection

C6 cells transient transfection was carried out using the calcium phosphate co-precipitation method. After 24 hours, cells were transfected with 5 µg of wild-type Opa-1 (WT Opa-1), mutant Q297V Opa-1 (both kindly provided from Dr. Donald D. Newmeyer, La Jolla Institute for Allergy and Immunology, USA) or pcDNA3 empty vector (gift from Dr. Elba Vazquez, Department of Biological Chemistry, Faculty of Exact and Natural Sciences, University of Buenos Aires, IQUIBICEN-CONICET, Argentina) in the presence of 10% FBS. After 5 hours, the medium was replaced by fresh grown medium and 24 hours post-transfection, cells were exposed to 750 µM MnCl_2_ during 24 hours in serum free medium. The transfection efficiency (∼60%) was determined by the percentage of pcDNA3-GFP transfected cells scored by fluorescence microscopy.

### Drp-1 Silencing

Cells were transfected for 48hs with Drp-1 siRNA or control siRNA using Lipofectamine 2000 according to the protocol provided by Dharmacon Thermo Scientific. For all experiments, the siRNAs were transfected at a final concentration of 20 nM). Silencing efficiency was evaluated by Drp-1 immunoblotting.

### Assessment of Cell Viability by MTT Assay

The conversion of the dye MTT to formazan by mitochondrial dehydrogenases was used as an index of cell viability according to the protocol previously described [21] with slight modifications [19]. After different treatments, MTT was added to cultures at a final concentration of 0.125 mg/ml and incubated for 90 min at 37°C. Then, formazan was solubilized in 200 µL of DMSO. Absorbance was measured at 570 nm with background subtraction at 655 nm in a BIO-RAD Model 680 Benchmark microplate reader (BIO-RAD laboratories, Hercules, CA, USA) and the MTT reduction activity was expressed as a percentage of the control cells.

### Subcellular Fractionation

Mitochondrial enrichment was carried out according to previous reports [22]. After MnCl_2_ exposure, C6 cells were lysed in MSHE buffer (0.22 M mannitol, 0.07 M sucrose, 0.5 mM EGTA, 2 mM HEPES-KOH, pH 7.4) containing protease inhibitors (0.5 mM PMSF, 1.54 µM aprotinin and 63.86 µM benzamidine). The homogenate was centrifuged for 10 min at 700×g and the resulting supernatant was centrifuged at 7000×g for 20 min to obtain the enriched-mitochondrial pellet. The latter was washed once in 150 mM KCl to deplete mitochondria of surrounding cytosolic mRNAs and then centrifuged at 10000×g for 10 min. Finally, the mitochondrial pellet was suspended in MSHE.

### Western Blotting

Western blots were performed according to standard procedures. Cells were suspended in a lysis buffer (50 mM HEPES/0.1% Triton pH 7.0, 0.5 mM PMSF, 10 µg/ml aprotinin and 10 µg/ml benzamidine) and incubated for 30min at 4°C. After centrifugation (10000×g, 20 min, 4°C), protein concentration was determined using Bradford assay [23]. Equal amount of proteins (60–80 µg) from each treatment was separated on 7.5 and 10% SDS- polyacrylamide gel electrophoresis (SDS PAGE) and blotted onto nitrocellulose membranes (Hybond ECL, GE Healthcare, Piscataway, NJ). Transference efficiency was verified by staining the membrane with Ponceau Red. Non-specific binding sites were blocked by 5% non fat dried milk in TBS (150 mM NaCl in 50 mM Tris-HCl buffer pH 8) containing 0.1% SDS, for 90 min and then incubated with specific antibodies overnight at 4°C. The primary antibody reaction was followed by incubation for 1 h with horseradish peroxidase-conjugated secondary antibodies. All antibodies were diluted in TBST (150 mM NaCl, 0.05% Tween 20, in 50 mM Tris–HCl buffer pH 8) with 3% non fat milk. The following primary and secondary antibodies were used: mouse anti- β-Actin (C4): sc-47778 (1∶10000), rabbit IgG-HRP: sc-2030 (1∶1000), mouse IgG-HRP: sc-2031 (1∶1000) were from Santa Cruz Biotechnology Inc. (Santa Cruz, CA, USA). Mouse anti- complex III subunit core 1-OxPhos (459140) (1∶5000) was obtained from Molecular Probes (Invitrogen, Eugene, OR, USA) and mouse anti- Opa-1 (612607) (1∶1000) and mouse anti- Drp-1 (611113) (1∶400) were from BD Pharmingen (San Diego, CA, USA). Immunoreactive bands were detected by chemiluminescence using ECL detection reagents. Inmunocomplexes were detected by using the LAS 1000 plus Image Analyser (Fuji, Tokyo, Japan) and the quantitative changes in protein levels were evaluated with the Image J software (NIH). The molecular weight of proteins was estimated by electrophoresis of protein markers (Fermentas Life Sciences, CA, USA). To confirm equal protein loading in each lane, antibodies were stripped from the membranes with stripping buffer (15% H_2_O_2_ in TBS) and reprobed with a loading control.

### Detection of Apoptotic Cells by Fluorescence Microscopy

Nuclear morphology analysis was performed by Hoechst 33258 staining [19]. In brief, C6 cells were sub-cultured on glass coverslips in 12- well plates at a density of 6×10^4^ cells/well. After treatments, cells were washed with PBS and fixed with 4% paraformaldehyde for 20 min at RT. Cells were washed twice with PBS, stained with Hoechst 33258 (1 µg/ml) and washed again with PBS. Image were analysed with an Olympus IX71 microscope equipped with objective lens 100X/1.65 oil (λ_ex_: 350/50 nm; λ_em_: 460/50 nm) (Olympus Corporation, Tokyo, Japan). Images were captured with a Hamamatsu Photonics ORCA-ER camera (Hamamatsu Photonics K.K., Systems Division, Hamamatsu, Japan). Digital pictures were analyzed and assembled using Adobe Photoshop 7.0 software. Uniformly stained and condensed and fragmented nuclei were scored as healthy (normal) and apoptotic cells respectively.

### Analysis of Mitochondrial Membrane Potential and Morphology

After exposure, cells were washed twice with PBS and incubated with the cell-permeant mitochondria-specific red fluorescent probe MitoTracker Red CMXRos at a final concentration of 75 nM in serum free-culture medium for 30 min at 37°C. Afterwards, cells were washed twice with PBS and fixed with 4% paraformaldehyde (20 min at RT). Finally, cells were washed with PBS and mounted on glass slides. Samples were examined under a fluorescence microscope Olympus IX71 (Olympus Corporation, Tokyo, Japan) equipped with objective lens 60X/1.43 oil (λ_ex_: 543/20 nm; λ_em_: 593/40 nm). Images were captured with a Hamamatsu Photonics ORCA-ER camera (Hamamatsu Photonics K.K., Systems Division, Hamamatsu, Japan). Digital images were optimized for contrast and brightness using Adobe Photoshop 7.0 Software and 3D images reconstructions were performed using the Huygens Deconvolution Software (Scientific Volume Imaging BV, Hilversum, Netherlands). To quantify the different mitochondrial morphologies, 200 cells/sample were scored and classified as cells exhibiting tubular (normal), intermediate (tubular with swollen regions) and fragmented (small and globular) mitochondria according to [19]. Cells classified as “loss of Δψm” refers to those showing nuclear condensation and loss of Δψm.

### Immunocytochemical Staining

C6 cells were cultured on glass coverslips in 24-well plates as described above. After treatments, cells were washed twice with PBS and incubated with MitoTracker Red CMXRos as previously described. Cells were washed twice with PBS, permeabilized with 0.25% Triton X-100 in PBS for 10min at RT, washed three times with PBS and incubated in a 1% bovine serum albumin (BSA)-PBST (137 mM NaCl; 2.68 mM KCl; 10 mM Na_2_HPO_4_; 1.76 mM KH_2_PO_4_; 0.05% Tween 20; pH 7.4) for 30 min at RT. The primary antibodies used were goat anti- Opa-1 (C-15): sc-30573 (Santa Cruz Biotechnology Inc., Santa Cruz, CA, USA) (1∶50) and anti- Drp-1 (611113) (BD Pharmingen, San Diego, CA, USA). Both antibodies were incubated for 48 h at 4°C and then conjugated for detection with Alexa fluor 488 anti-goat (A-11055) and Alexa fluor 488 anti- mouse IgG (A-11001) (Molecular Probes, Invitrogen, Eugene, OR, USA) respectively. Samples were examined under a confocal fluorescence microscope Nikon Eclipse E800 C1 (Nikon Instech Co., Ltd., Karagawa, Japan), equipped with objective lens Nikon Plan Apo 60X/1.40 Oil (Mitotracker Red: λ_ex_: 544 nm; λ_em_: 570-LP nm; Alexa 488: λ_ex_: 488 nm; λ_em_: 515–530 nm). Digital pictures were analyzed and assembled using Image J software (NIH). Co-localization index was determined based on Pearson’s and Manders’ correlation coefficients. A co-localization index of 1.0 indicates 100% of merge (yellow-orange) between the analyzed protein (green) and mitochondria (red). Data were rendered as the average co-localization index obtained by analysis of combining data from at least two independent experiments. In each experiment 15 cells were analyzed from at least four randomly chosen fields for each treatment as previously described [24].

### In vivo Manganese Treatment

Before MnCl_2_ administration, rats (n = 8) were deeply anesthetized with a Xylazine (20 mg/kg)/Ketamine (50 mg/kg) cocktail and were positioned in a stereotaxic frame (David Kopf Stereotaxic Instruments, Tujunga, CA, USA) with the incisor bar at the level of the ear. MnCl_2_ dissolved in sterile distilled water (1 µmol MnCl_2_) was injected into the left striatum and saline into the right one using the following coordinates: A = −0.2, L = ±3, V = −5 to the bregma, according to Paxinos and Watson atlas [25]. Infusions were performed at an average flow rate of 1 µl/40 sec and the cannula remained in situ for 2 min after the completion of each infusion.

### Immunohistochemical Staining

Animals treated with MnCl_2_ for 24 hours or 7 days were perfused with 4% paraformaldehyde and brains were dissected. Coronal sections (50 µm) were cut through the entire brain using a vibrating microtome Integraslice 7550PSDS (Campden Instruments, Loughborough, United Kingdom). Sections were stored in a cryoprotectant solution (25% glycerol, 25% ethylene glycol, 50% sodium phosphate buffer 0.1 M pH 7.4) at −20°C until use. All immunohistochemical stainings were performed on free-floating sections from 4 rats per group. Brain sections were placed in 24-well plates and washed 3 times with PBS (10 min). To block endogenous peroxidase activity, sections were exposed for 10 min to 3% H_2_O_2_ in a solution of 100% methanol: PBS (1∶1). Unspecific antigenic sites were blocked for 30 min at 37°C treatment with PBS containing 0.1% Triton X-100 and 10% horse serum. Then, sections were incubated (48 hours, 4°C) with the primary antibody goat anti- Opa-1 and mouse anti- Drp-1 both in PBS containing 0.1% Triton X-100 and 0.1% horse serum. Negative controls were performed by incubating brain sections under the same conditions without primary antibody (data not shown). Sections were washed 3 times with PBS and incubated with the corresponding biotinylated secondary antibody for 1 hour at RT followed by the ABC kit procedure. Detection was performed with 2 mM DAB and 0.5 mM H_2_O_2_ in 0.1 M Tris buffer at RT and brain sections was visualized by a Leica DM2000 microscope coupled with a Leica ICC50 camera (Leica Microsystems, Wetzlar, Germany).

### Nissl Staining

Morphological analysis of brain tissue was carried out using Nissl staining. This technique was performed on brain sections prepared using the same fixation and cutting protocol described above for immunohistochemical procedures. Cryoprotected sections were thoroughly rinsed in PBS, put on gelatin-treated slides and air-dried overnight. Then, slides were briefly washed in distilled water and then immersed in a 0.5% cresyl violet solution for 10min. After that, sections were dehydrated through a series of graded ethanol (70% 2×1 min each, 95% 2×1 min each and 100% 2×2 min each) and cleared in xylene (2×3 min each). Slides were coverslipped using Permount and samples were analyzed using a Leica DM2000 microscope coupled with a Leica ICC50 camera (Leica Microsystems, Wetzlar, Germany).

### Detection of Apoptotic Cells

Apoptotic cell death was determined on sections stained with Hoechst 33258. 3–4 slices from each animal were placed in 24-well plates, washed 3 times with PBS (10 min), stained with Hoechst 33258 solution (2.5 µg/ml in PBS) during 15 min and washed 3 times with PBS (10 min). Sections were mounted using Permount and observed under fluorescence microscope Olympus IX71 microscope equipped with objective lens 100X/1.65 oil (λ_ex_: 350/50 nm; λ_em_: 460/50 nm) (Olympus Corporation, Tokyo, Japan). Images were captured with a Hamamatsu Photonics ORCA-ER camera (Hamamatsu Photonics K.K., Systems Division, Hamamatsu, Japan).

### Statistical Analysis

Experiments were carried out at least in triplicate unless otherwise stated. Results are expressed as mean ± SEM values. Experimental comparisons between treatments were made by Student’s t- test and one- way ANOVA, followed by Student- Newman- Keuls post hoc test with statistical significance set at p<0.05. All analyses were carried out with GraphPad Prism 4 software (GraphPad Sofware, San Diego, CA, USA).

## Results

### Mn Induces Changes in the Expression Levels and Subcellular Localization of Opa-1 and Drp-1

Previous findings of our laboratory have demonstrated the involvement of the mitochondrial apoptotic pathway in rat C6 cells Mn-induced apoptosis [19]. In that report we showed that in addition to produce Δψm dissipation, Mn induces an imbalance in fusion/fission equilibrium resulting in a mitochondrial fragmentation enhancement. While Drp-1 is considered the fission master regulator, Opa-1 and Mfn-1/2 are required for Outer- Mitochondrial Membranes (OMM) fusion. Distinctly, Opa-1 is also crucial for the Inner-Mitochondrial Membranes (IMM) fusion and the maintenance of the proper mitochondrial cristae architecture. This evidence prompted us to perform studies aiming to elucidate the role of Opa-1 and Drp-1 in this experimental model.

**Figure 1:**
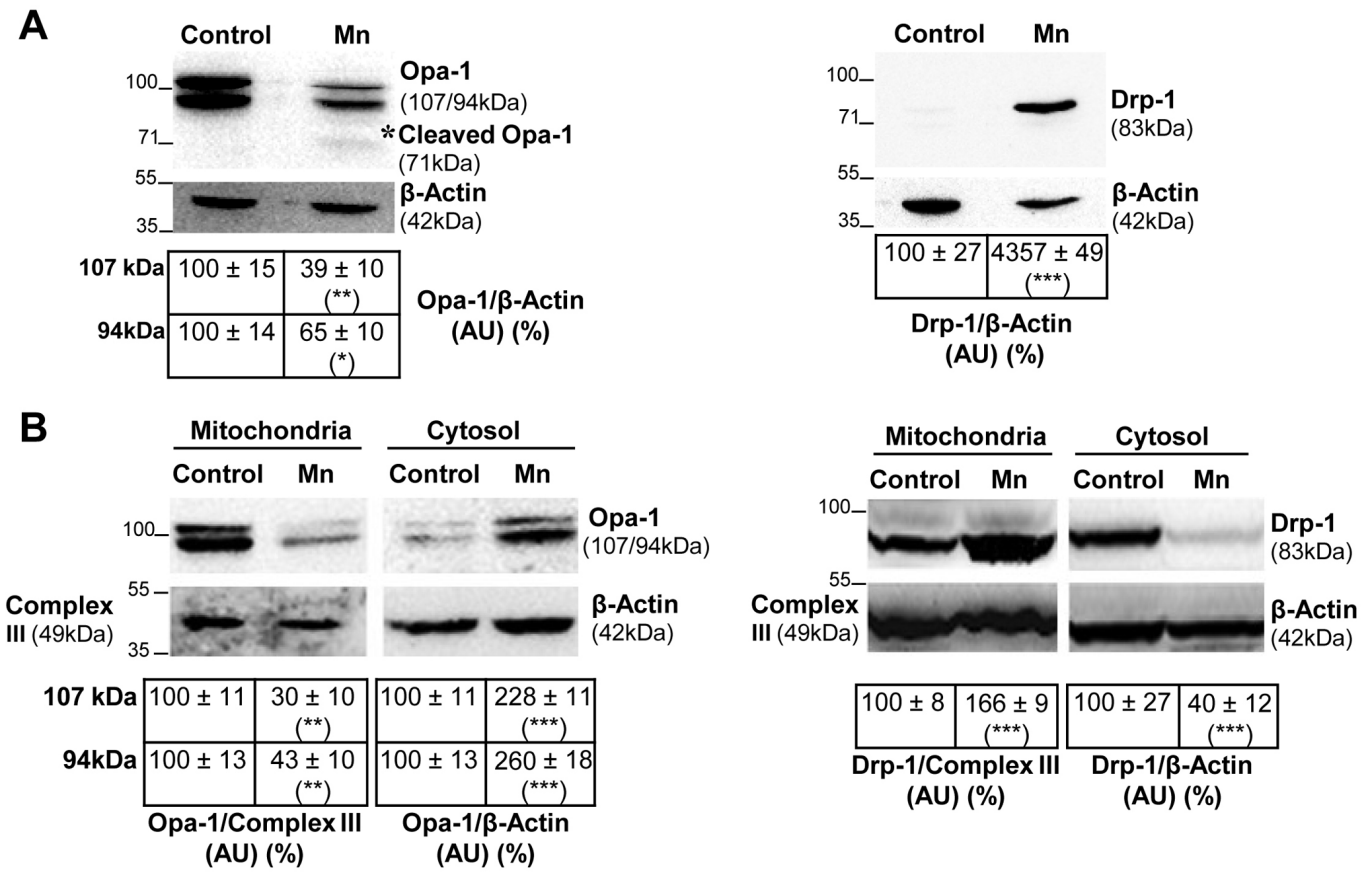
Altered expression and subcellular distribution of mitochondrial fusion/fission proteins in Mn-treated C6 cells. Opa-1 and Drp-1 expression levels. Monoclonal antibodies that recognize Opa-1 or Drp-1 were used for immunoblotting analysis of total cell lysates. Reprobing with an anti-β-Actin antibody was performed to normalize for protein loading. Signals were quantified with the Image J software. Images correspond to one representative experiment (n = 3). Results are expressed as a percentage of the respective control considered as 100%. Asterisk [*] denotes Opa-1 cleavage product with a MW ∼71 kDa (**A**); Enriched-mitochondrial and cytosolic fractions were subjected to immunoblotting procedure using antibodies that recognize Opa-1 or Drp-1 proteins. Reprobing with anti-Complex III subunit core 1- OxPhos (Complex III) and anti-β-Actin antibodies was performed to normalize for loading control. Images correspond to one representative experiment (n = 2). Results are expressed as a percentage of the respective control considered as 100% (**B**). Statistically significant differences between the controls and experimental groups are indicated by: *p<0.05, **p<0.01 and ***p<0.001 vs. control. AU: Arbitrary Units.

**Figure 2:**
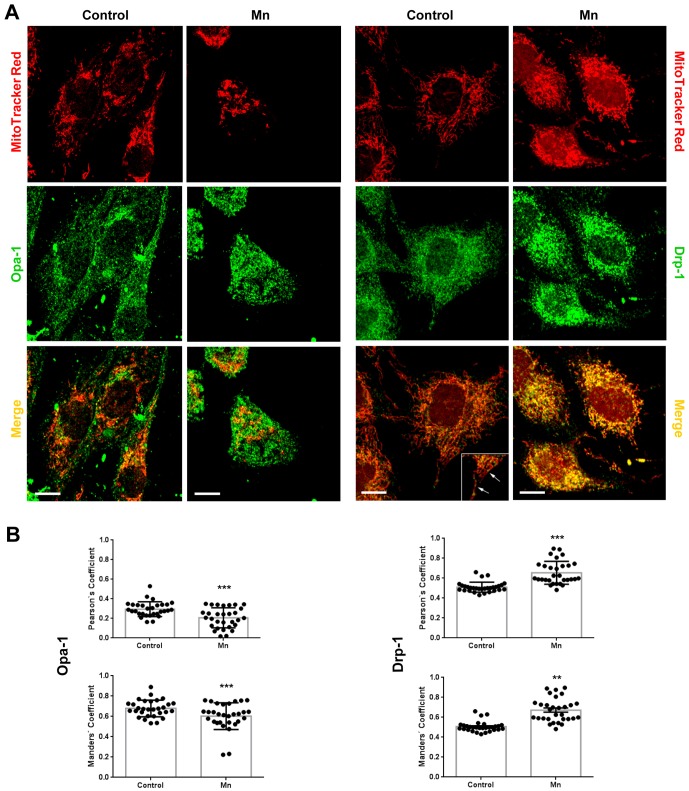
Opa-1 and Drp-1 immunocytochemistry. Representative confocal microscopy images obtained in separate experiments (n = 2) Scale bar: 10 µm (**A**); Scatter plots of Pearson’s and Manders’ coefficients. Statistical significance, **p<0.01 and ***p<0.001 vs. control (**B**).

First, we investigated the effect of Mn-exposure on Opa-1 and Drp-1 expression levels in total glial lysates (Fig. 1A). Immunoblot analysis of Mn exposed cells revealed significant changes in Opa-1 and Drp-1 expression levels in comparison with controls. Opa-1 was observed as a doublet band with estimated molecular weights (MW) of ∼107 and ∼94 kDa. Mn exposure decreased both bands expression levels (61% and 35%, p<0.05 respectively). In addition we also observed the appearance of a lower MW fragment (∼71 kDa) (Fig. 1A asterisk). Unlike Opa-1, Drp-1 levels were dramatically increased (∼43- fold; p<0.001) in cell lysates under Mn-treatment in comparison with control. The MW estimated for Drp-1 was 83 kDa in accordance with the previously established weight for brain isoform [26]. Increasing evidence indicates that both the release of Opa-1 into the cytosol and the translocation of Drp-1 to the mitochondria are crucial events for the prominent increase in mitochondrial fragmentation observed during apoptosis [14], [26]–[28]. Based on the preceding results, we next studied the subcellular localization of Opa-1 and Drp-1 in Mn-treated cells (Fig. 1B). As we expected, Mn exposure resulted in decreased levels of Opa-1 high MW forms in the mitochondria (70% and 57%; p<0.01) with a concomitant increase in cytosolic levels (128% and 160%; p<0.001). Therefore, these events both indicate the release of this protein from the mitochondria. On the other hand, Mn exposure increased the mitochondrial levels of Drp-1 (66%, p<0.001) in parallel with a significant reduction in the cytosolic expression of this protein (60%, p<0.001). To confirm these results, we carried out immunocytochemical studies (Fig. 2A). In control cells, Opa-1 staining co-localized with Mitotracker Red CMXRos, whereas the release of this protein to the cytoplasm took place in Mn-exposed cells (Pearson and Manders co-localization coefficients for Mn treatment: 0.17±0.02 and 0.56±0.02 respectively; p<0.001, Fig. 2B). In line with these results, control cells exhibited the presence of Drp-1 in both the cytoplasm and specific spots or “foci” along the mitochondrial tubular network, whereas in cells incubated with Mn, a larger amount of Drp-1 than the one measured in control cells is recruited to mitochondria. This data confirms Drp-1 translocation to the mitochondria (Pearson and Manders co-localization coefficients for Mn treatment: 0.87±0.02 and 0.65±0.03 correspondingly; p<0.01 and p<0.001 respectively, Fig. 2B). Results obtained point to Opa-1 and Drp-1 as possible players in the occurrence of increased mitochondrial fragmentation triggered by Mn.

### Opa-1 Over-expression Confers Resistance to Apoptosis Triggered by Mn

**Figure 3:**
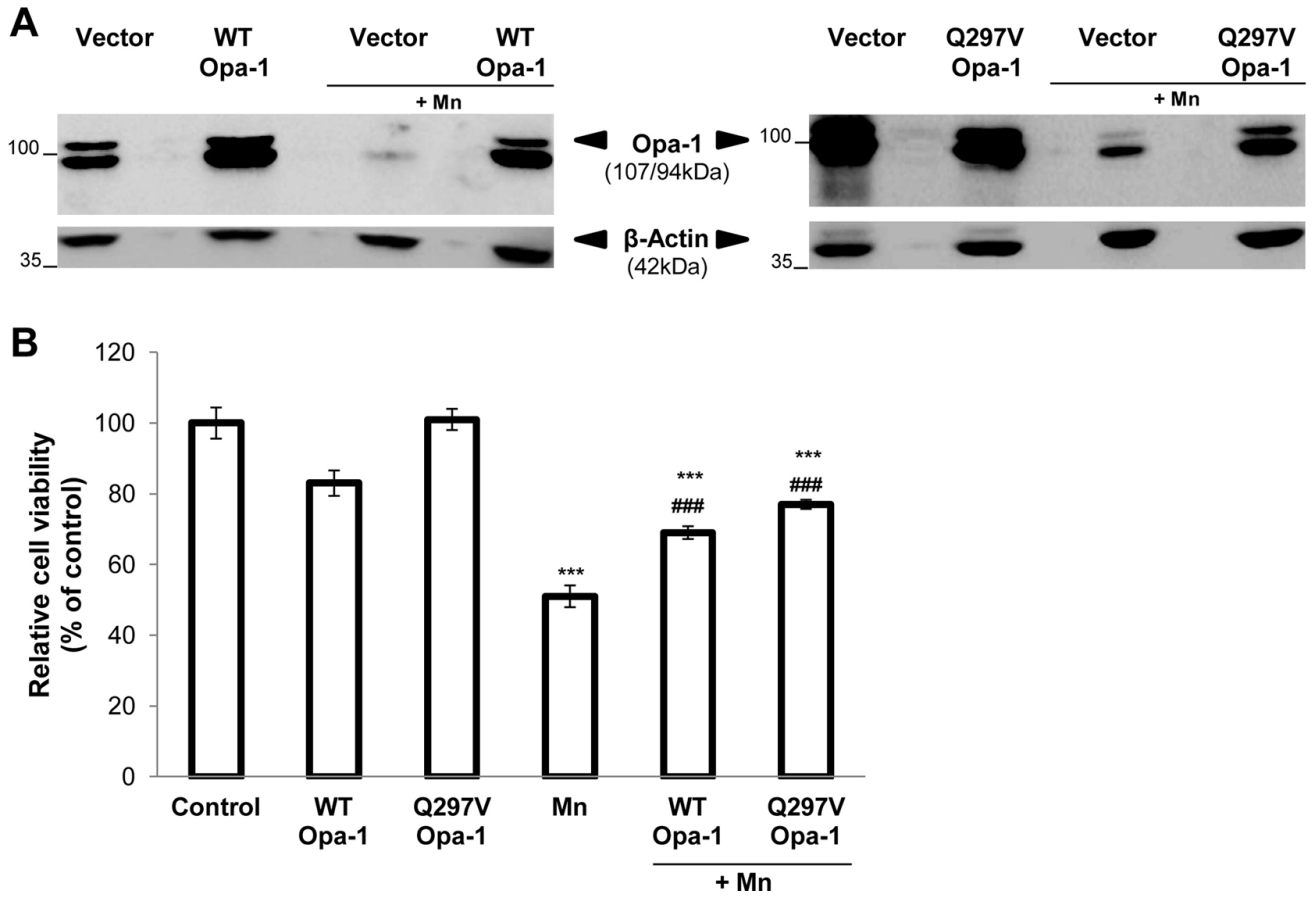
WT and Q297V Opa-1 (disassembly-resistant mutant) over- expression result in cell death reduction. C6 cells were transiently transfected with empty pcDNA3 vector (control) or vectors encoding WT or Q297V Opa-1, grown for 24 hours and then exposed to Mn for additional 24 hours. Western Blot of total lysates from transfected cells (**A**); Cell viability determined by MTT assay (**B**). Statistical significance, ***p<0.001 vs. control and ###p<0.001 vs. Mn.

**Figure 4:**
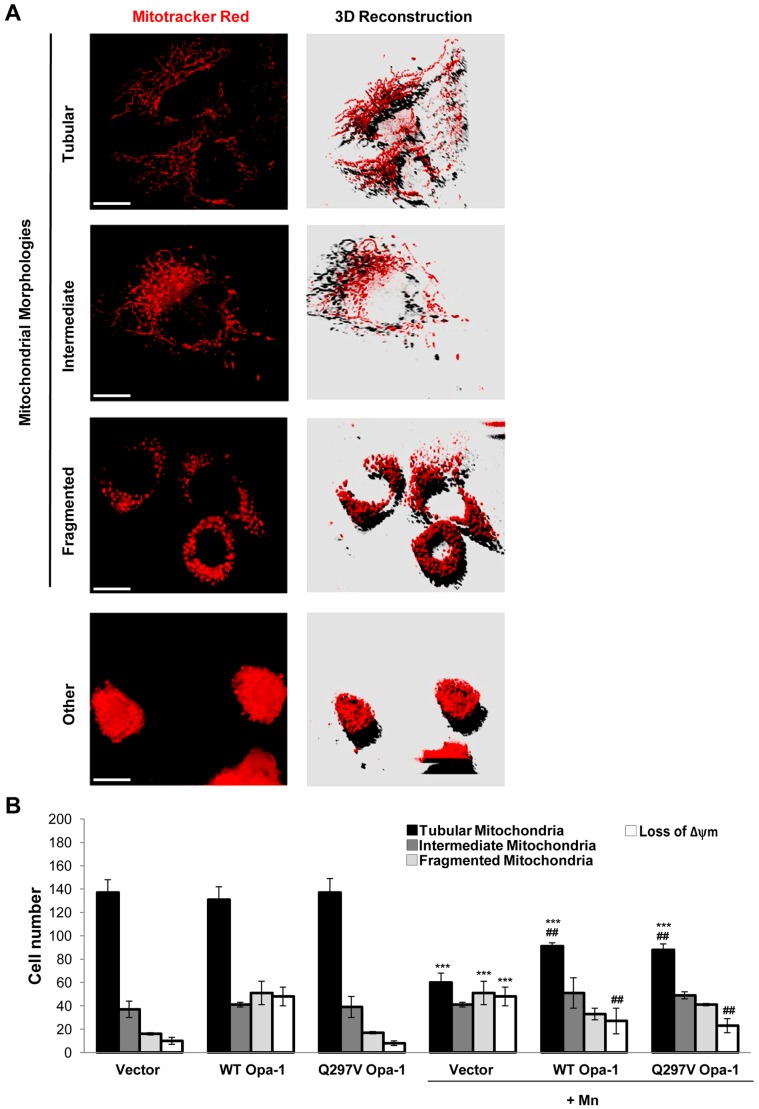
WT and Q297V Opa-1 over-expression maintain the mitochondrial tubular network. Mitochondria were visualized with MitoTracker Red CMXRos (75 nM) under a fluorescence microscope. Representative images acquired and deconvoluted are shown**.** Scale bar: 10 µm. Three categories of cells exhibiting different mitochondrial morphology were scored: tubular mitochondria (normal), intermediate mitochondria (filamentous with swelling regions) and fragmented (globular). Cells classified as “loss of Δψm” refers to those exhibiting nuclear condensation and Δψm collapse (**A**); 200 cells/sample were counted and classified according to item A (**B**). Two independent experiments were conducted. Statistical significance, ***p<0.001 vs. control and ##p<0.01 vs. Mn.

**Figure 5:**
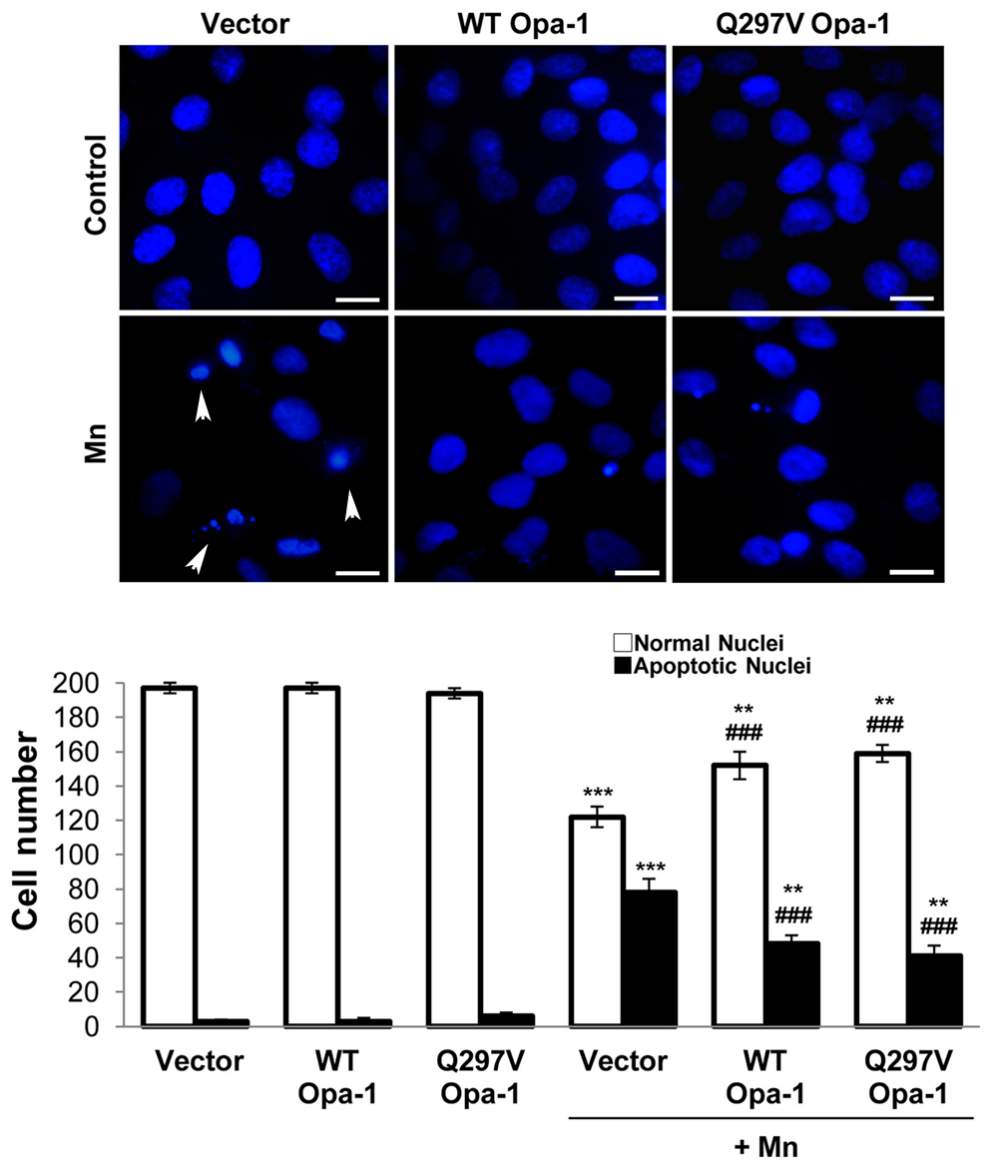
WT and Q297V Opa-1 over-expression prevent apoptotic nuclei appearance. Normal and apoptotic (condensed and fragmented chromatin) (arrowheads) nuclei were detected with Hoechst 33258 (1 µg/ml) by fluorescence microscopy and 200 cells/sample were scored. Scale bar: 10 µm. Two independent experiments were conducted. Statistical significance, **p<0.01 and ***p<0.001 vs. control; ###p<0.001 vs. Mn.

To reveal the possible role of Opa-1 in the Mn-apoptotic signaling pathway we analyzed the effect of transfecting C6 cells with either WT or Q297V Opa-1 cDNAs on cell viability, mitochondrial morphology and apoptotic parameters. Q297V Opa-1 mutant is characterized by the substitution of glutamine 297 for valine, in the GTPase domain (295–470). The resulting protein lacks of GTPase activity but exhibits gain of another activity, which consists in stabilizing Opa-1 complexes. Furthermore, it has been proposed that this mutant should mimic the GTP-bound form of Opa-1, thereby preventing the disassembly of the mitochondrial cristae complexes structure [29]–[31]. In accordance with previous reports employing transient transfected cells [31], [32] the immunoblots showed a modest difference in the expression levels of exogenous and endogenous Opa-1 proteins (Fig. 3A). Despite this expression pattern, both cDNAs were able to markedly restore the Opa-1 levels that have been almost totally reduced by Mn treatment. We next analyzed the effect of Opa-1 overexpression on cell viability through MTT assay. Transfection with WT and Q297V Opa-1 could partially prevent cell death (20% and 25% respectively; p<0.001) (Fig. 3B). Employing Mitotracker Red CMXRos staining we evaluated the mitochondrial network morphology and Δψm dissipation. For the former analysis we generated a 3D image reconstruction and volumetric rendering corresponding to samples visualized with fluorescence microscopy. Tubular structures that move in and out of the focal plane can be easily mistaken for individual rod or spherical organelles in conventional imaging [33], [34]. Therefore, the stacks acquisition of mitochondrial images along the z-axis of the entire cell gave us a more detailed visualization and quality of the morphological changes that occur in mitochondria exposed to Mn and also, allowed us to establish a more precise morphological classification of these organelles (Fig. 4A). When different types of mitochondrial morphologies were scored, we found that WT and Q297V Opa-1 exerted a protective effect on mitochondrial network. In fact, Opa-1 transfected cells showed an increase in the number of cells with preserved tubular mitochondria (22% and 20%; p<0.01) and a diminished number of cells presenting loss of Δψm compared to control Mn-treated cells (35% and 40%; p<0.01) (Fig. 4B). Finally, we analyzed nuclei morphology by Hoechst 33258 staining and fluorescence microscopy. Uniformly stained nuclei were scored as healthy, viable cells whereas those exhibiting condensed and fragmented chromatin were classified as apoptotic cells (Fig. 5). Control cells transfected with empty pcDNA3 vector showed normal nuclei with homogeneous staining. In contrast, cells exposed to Mn showed an increase in apoptotic nuclei according to previous results of our group [19]. This effect was prevented (25%; p<0.01) in cells over-expressing WT or Q297V Opa-1. Altogether, these results confirm our previous findings [19] demonstrating that Mn induces an imbalance in the fusion/fission mitochondrial equilibrium resulting in a mitochondrial fragmentation exacerbation. The increase in the number of cells presenting tubular mitochondria in WT and Q297V Opa-1 over-expressed Mn-treated cells confirms that Opa-1 is involved in mitochondria fusion in our model. Given that Q297V Opa-1 mutant inhibits the disruption of Opa-1 complexes our results suggest that Mn may produce the processing and disassembly of Opa-1 complexes which contributes to the progression of the apoptotic cascade.

### Effects of CsA on Mn-induced Depolarization, Opa-1 Processing, Drp-1 Translocation and Mitochondrial Fragmentation

**Figure 6:**
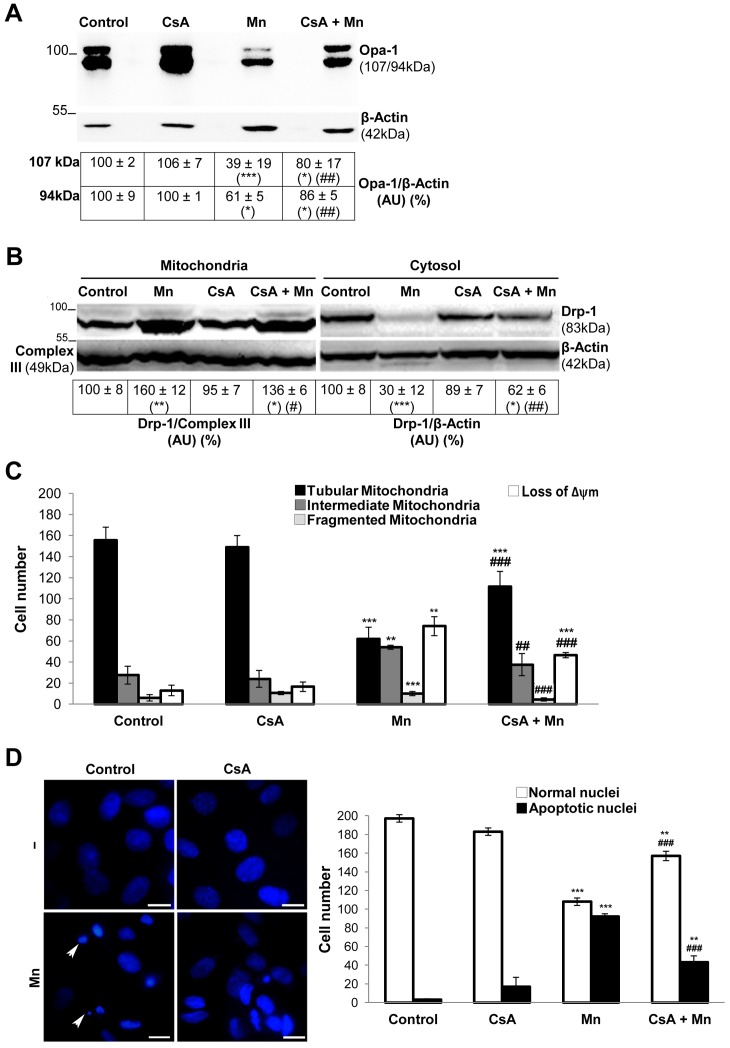
CsA prevents Mn-induced Opa-1 cleavage and Drp-1 translocation to mitochondria. Opa-1 expression levels in total cell lysates. Anti-β- Actin antibody was used as a loading control (**A**); Drp-1 expression levels in enriched-mitochondrial and cytosolic fractions. Membranes were stripped and reprobed for anti-Complex III subunit core 1- OxPhos (Complex III) I and anti-β- Actin as loading control (**B**); **CsA diminishes Mn-induced mitochondrial fragmentation, loss of Δψm and apoptotic nuclei appeareance.** Quantification of mitochondria (**C**) and nuclei morphology (**D**) classified according to Figs. 4, 5. Scale bar: 10 µm. Three independent experiments were conducted. Statistical significance, **p<0.01 and ***p<0.001 vs. control; ##p<0.01 vs. Mn. Arrowheads: apoptotic (condensed and fragmented chromatin) nuclei.

The MPTP opening is a process that engages the IMM and the OMM and triggers a second wave of reactive oxygen species (ROS) generation as a result of the decoupling of the electron transport chain [35]. Previous results of our group have demonstrated that Mn induces ROS generation which results to be lethal for C6 cells [19]. Furthermore, employing Cyclosporin A (CsA), an inhibitor of MPTP opening [36], we found that MPTP is involved in the cytotoxic effects displayed by Mn on mitochondria. In order to determine whether the MPTP opening had an impact on Opa-1 processing, we pre-incubated cells with CsA (1 µM) and evaluated its effect on Opa-1 levels by western blotting (Fig. 6A). CsA, partially prevented Opa-1 cleavage (41% and 25% for 107 and 94 kDa isoforms respectively; p<0.01).This data suggest that MPTP opening triggered by Mn contributes to Opa-1 processing.

Drp-1 dephosphorylation by the cytosolic Ca^2+^-dependent phosphatase calcineurin regulates its translocation to the mitochondria, a process which contributes to fragmentation, OMM permeabilization and ensuing apoptosis [37], [38].

Mn alters intracellular Ca^2+^ homeostasis [39] which could in turn induce calcineurin activation. Taking into account that CsA blocks calcineurin action, we analyzed the effect of CsA on Drp-1 translocation to mitochondria (Fig. 6B). Cells pre-incubated with CsA and exposed to Mn showed an increased expression of Drp-1 in the cytosolic fraction (32%; p<0.01) with a concomitant decrease in its mitochondrial levels (24%; p<0.05) in comparison with Mn-treated cells. These results suggested that CsA could interfere with Drp-1 Mn-induced translocation to mitochondria. Hence, we next examined the effect of CsA on Mn-induced mitochondrial fragmentation in C6 cells. In addition to preventing the mitochondrial Δψm disruption (38%, p<0.001), CsA could diminish the mitochondrial network dismantling (Fig. 6C). In fact, more cells with tubular (35%; p<0.001) and less with intermediate and fragmented mitochondria (66% and 40%; p<0.001) were scored in cells pre-incubated with CsA and then exposed to Mn. Moreover, CsA ultimately inhibited apoptosis (24%; p<0.001) demonstrated by a decrease in the amount of condensed and fragmented nuclei stained with Hoechst 33258 (Fig. 6D).

Altogether these data suggest that CsA exerts its effects at different levels, preventing MPTP opening, Opa-1 processing and Drp-1 translocation to mitochondria. As a consequence, CsA protects Mn-exposed cells from mitochondrial disruption and apoptosis.

### Pharmacological and Genetic Inhibition of Drp-1 Protects Against Mn-induced Apoptosis

To further confirm the role of Drp-1 in Mn-induced apoptosis we conducted experiments employing Mdivi-1, a small specific inhibitor of Drp-1, and a mixture of SMART selection-designed Drp-1 targeted siRNAs.

**Figure 7:**
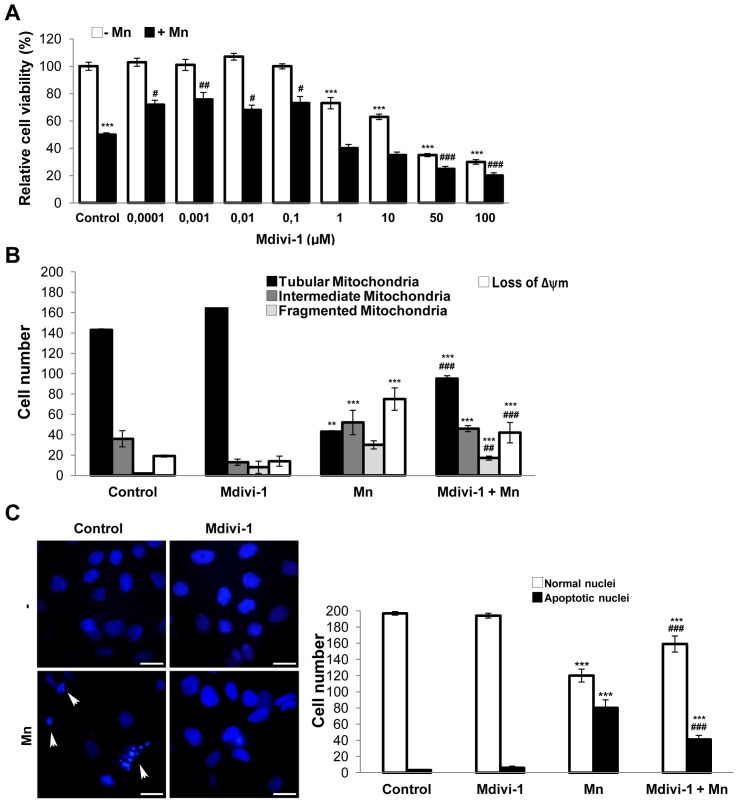
Mdivi-1 protects from Mn injury. Viability was measured by MTT assay (**A**); Quantification of mitochondrial morphology and Δψm dissipation analysis using MitoTracker Red CMXRos (75 nM) (**B**); Apoptotic nuclei were determined by staining with Hoechst 33258 (arrowheads). Scale bar: 10 µm (**C**). Results are average of three individual experiments. Statistical significance, **p<0.01 and ***p<0.001 vs. control; #p<0.05, ##p<0.01 and ###p<0.001 vs. Mn.

First, we performed a dose-response curve in the concentration range of 0.0001–100 µM Mdivi-1 in the presence and absence of Mn (Fig. 7A). Concentrations in the range of 0.0001–0.1 µM were protective against cell death whereas those higher than 1 µM resulted cytotoxic probably due to undesirable fission inhibition. We choose 0.001 µM as the concentration to be used in future experiments. Using Mitotracker Red CMXRos staining, we determined that cultures pre-incubated with 0.001 µM Mdivi-1 showed an increase in cells exhibiting tubular mitochondria (30%; p<0.001) and a decrease number of cells with fragmented mitochondria and Δψm loss (43% and 35% respectively; p<0.001) in comparison with those treated with Mn (Fig. 7B). In addition, Mdivi-1 attenuated the effect of Mn by preventing nuclear condensation and fragmentation (32%; p<0.001) (Fig. 7C).

**Figure 8:**
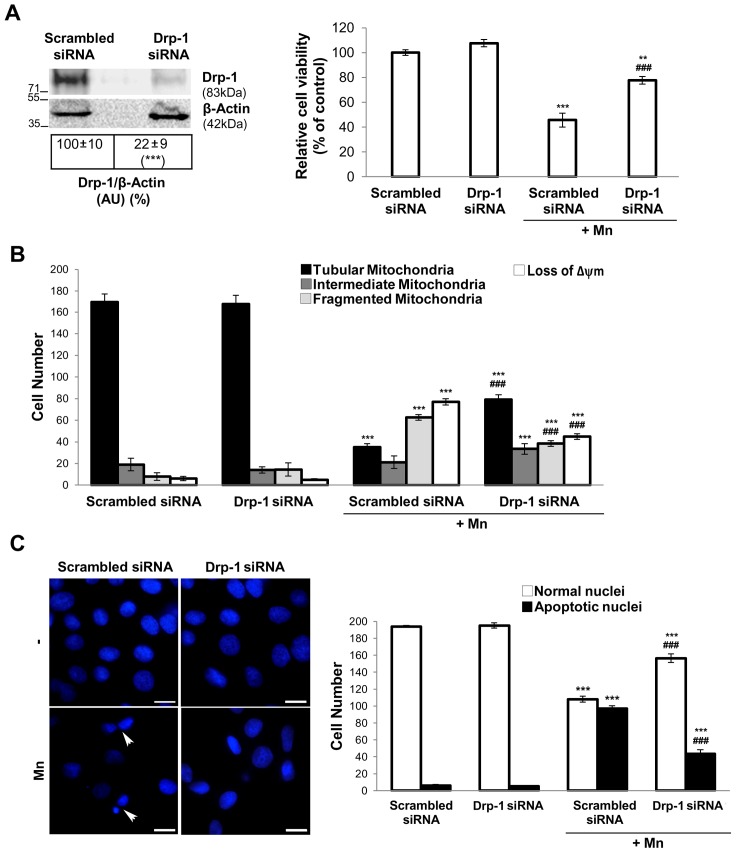
Drp-1 siRNA prevents Mn damage. Western blot analysis of Drp-1 protein in C6 cells transfected with pooled Drp-1 or control siRNA for 48 hours. Reprobing with an anti-β-Actin antibody was performed to normalize for protein loading. Results are expressed as a percentage of the respective control considered as 100%. Viability was measured by MTT assay (**A**); Quantification of mitochondrial morphology and Δψm dissipation analysis using MitoTracker Red CMXRos (75 nM) (**B**); Apoptotic nuclei were determined by Hoechst 33258 staining (arrowheads). Scale bar: 10 µm (**C**). Results are average of three individual experiments. Statistical significance, **p<0.01 and ***p<0.001 vs. control; ###p<0.001 vs. Mn.

Drp-1 siRNA significantly reduced Drp-1 protein levels (78%; p<0.001) and partially prevented Mn-induced cell death (32%; p<0.001) (Fig. 8A). In addition, cells pre-treated with Drp-1 siRNA exhibited increased tubular mitochondria (28%; p<0.01), decreased fragmentation and reduced loss of Δψm compared to scrambled siRNA Mn-exposed cells (60% and 32%; p<0.001) (Fig. 8B). Moreover, Drp-1 silencing partially prevented the appearance of apoptotic nuclei (29%; p<0.001) (Fig. 8C).

Altogether these results demonstrated that Drp-1 protein contributes to apoptosis in Mn-treated C6 cells.

### Mn-induced Striatal Damage with Apoptotic Characteristics

**Figure 9:**
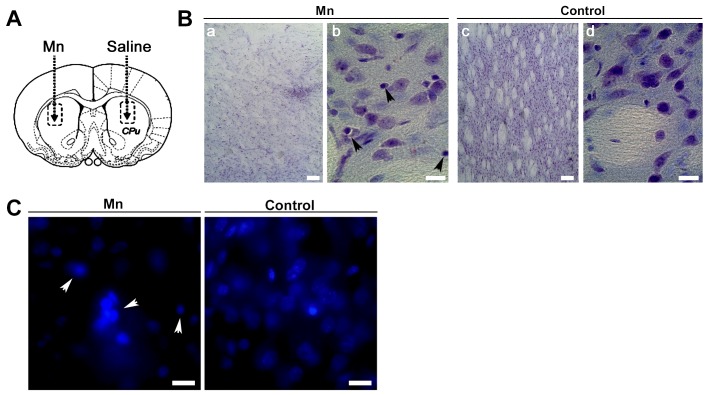
Mn induced damage into rat striatal tissue (**A**). Rats were injected into the striatum with 1 µmol Mn (left) and saline solution (right), as indicated with dotted lines. **Nissl staining on vibratome brain sections containing striatum from Mn-treated (a,b) and control (c,d) rats** (**B**). Magnification: 10X (a,c) and 40X (b,d); **Nuclei staining** (**C**). Arrowheads: cells with shrunken shape and pyknotic nuclei. Magnification: 100X. Samples correspond to sections from rats receiving a single striatum injection of Mn and euthanized 7 days after. Scale bar: 10 µm.

To deeper investigate the effect of Mn on cell death we performed experiments employing a rat model of Mn intoxication. In these studies, we injected 1 µmol Mn into the right striatum (Fig. 9A). After 24 hours or 7 days post injection, none of the animals showed signs of locomotive failure. Nissl-stained sections of injected striatal regions revealed no evident damage in animals exposed to Mn for 24 hours (data not shown). In contrast, lesions with marked cellular loss and presence of pyknotic nuclei became apparent in treated rats after 7 days (Fig. 9B, panels a, b). These results were confirmed employing the fluorescent probe Hoechst 33258 (Fig. 9C).

### Altered Expression Levels of Mitochondrial Fusion and Fission Proteins after Striatal Mn-injection

**Figure 10:**
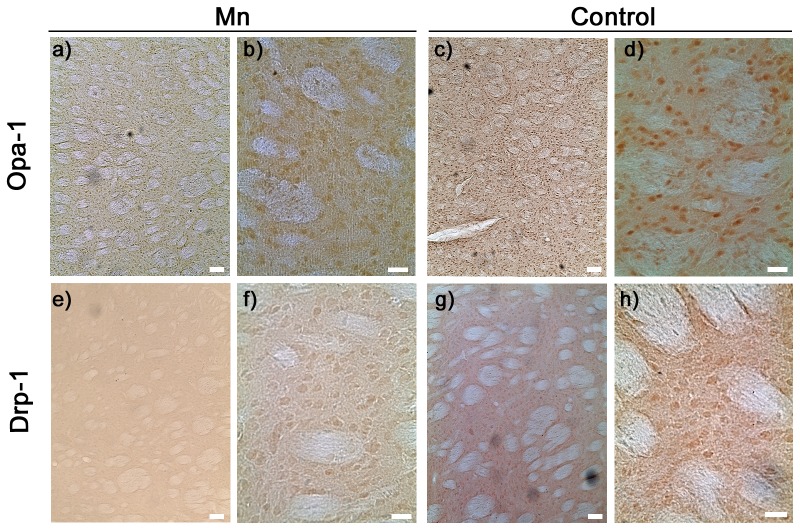
Opa-1 and Drp-1 expression in rat striatum. Representative immunohistochemistry images of Opa-1 (upper panel) and Drp-1 (lower panel). Samples correspond to sections from rats receiving a single (left) striatum injection of Mn (a,b,e,f) or a single (right) striatum injection of saline (c,d,g,h) and euthanized 7 days after. Magnification: 10X (a,c,e,g) and 40X (b,d,f,h). Scale bar: 10 µm.

We next studied Opa-1 and Drp-1 expression levels in striatal tissue from 7-days Mn treated rats. Immunohistochemistry revealed a reduced immunoreactivity of Opa-1 in accordance with our *in vitro* experiments (Fig. 10, panels a, b). Interestingly, we detected a reduced Drp-1 immunoreactivity in comparison with control striatum (Fig. 10, panels e, f). At any rate, these data suggest that an impaired mitochondrial fusion and fission balance is implicated in Mn-induced toxicity *in vivo*.

## Discussion

Mitochondria are morphologically dynamic organelles continuously ongoing fusion and fission processes forming from interconnecting tubular networks to small isolated organelles [10]. During apoptosis, mitochondrial networks become extensively fragmented resulting in an increased amount of smaller organelles. This event takes place usually upstream of caspases activation and close in time to Bax translocation, mitochondrial outer membrane permeabilization (MOMP) and cytochrome c release [40], indicating that apoptosis and mitochondrial morphology changes are linked processes.

Employing Mn exposed C6 cells as an *in vitro* model for investigating molecular mechanisms involved in Mn toxicity, our group has demonstrated that in addition to produce Δψm dissipation, Mn induces an imbalance in fusion/fission equilibrium resulting in an enhancement of mitochondrial fragmentation [19]. These findings based on the analysis of different mitochondrial morphologies indicated that in C6 cells Mn-induced apoptosis, the mitochondrial dynamics is altered in accordance with previous reports in models of different neurodegenerative diseases like PD, Huntington Disease, AD, Amyotrophic Lateral Sclerosis and Charcot-Marie-Tooth [14], [15], [41].

The identification of the proteins involved in the regulation and maintenance of mitochondrial morphology has revealed a critical role of mitochondrial dynamics in cell physiology. As a consequence, defects in fusion and fission proteins result in cellular homeostasis deregulation, apoptosis and neurodegeneration [42]. While a number of key mitochondria-shaping proteins have been identified, the precise mechanisms which govern their activity remain elusive. In this work we investigate the role of Opa-1 and Drp-1 in C6 cells Mn-induced apoptosis. Western blot analysis of Opa-1 showed the presence of two bands of 107 and 94 kDa (Fig. 1A). This high MW doublet band has already been reported by several groups [43]–[48] in cells of nervous origin. These authors described in different experimental models the presence of two bands whose MW’s fluctuated between 80–100 kDa. Particularly, the occurrence of different bands in heart, liver and skeletal muscle rat tissues, human lymphocytes and HeLa cells has suggested that the number and MW of bands detected by western blot analysis is dependent of tissue and specie under study [43]. On the other hand, our results revealed that Mn induced the Opa-1 cleavage judging by the appearance of the ∼71 kDa fragment (Fig. 1A). This band corresponding to a cleavage product without GTP-ase activity was only described by Linseman’s group in rat primary cerebellar granule neurons treated under conditions that trigger the intrinsic apoptotic pathway [46], [48]. Obviously, this band of ∼71 kDa is a difficult product to detect and in fact we could not achieve reproducibility on its detection throughout the experiments. This Opa-1 cleavage would explain the increased mitochondrial fragmentation observed in Mn-exposed cells. However, an effect of decreased high MW Opa-1 expression levels independently of Opa-1 cleavage may not be discarded. In previous reports, a reduction in the high MW Opa-1 isoforms levels by the action of apoptotic stimuli-activated proteases has been described [49], [50].

The subcellular localization analysis of Opa-1 showed the release of the high MW Opa-1 isoforms from the mitochondria to the cytosol in Mn-treated cells (Fig. 1B) possibly contributing to the increase in mitochondrial fragmentation induced by Mn (Fig. 4A). Results reporting mitochondrial fission and cristae architecture alterations were also found in primary cultures of retinal ganglion cells derived from mice suffering glaucoma. Furthermore, western blot analysis of subcellular fractions showed that high MW Opa-1 isoforms were cleaved into lower MW isoforms and both released to cytoplasm [45].

With the aim to establish the role of Opa-1 in Mn-induced apoptosis, C6 cells were transfected with WT and Q297V Opa-1 plasmids. According to previous reports [31], [32] this strategy would contribute to the Opa-1 oligomers stabilization preventing the dismantling of mitochondrial cristae structure. This latter group have reported that transfection of MEFs Afg3l2^−/−^ with WT or Q297V Opa-1 cDNAs resulted in a partial restoration of OPA1 L2 band (a high MW mouse OPA-1 isoform). In accordance, we showed that WT and Q297V Opa-1 transfection prevents high MW Opa-1 isoforms cleavage (Fig. 3A). When the role of Opa-1 in apoptosis was evaluated, we found that this was a bifunctional protein acting as an anti-apoptotic protein and fusion promoter in agreement with previous findings [51]. In fact, Opa-1 was able to prevent cell death (Fig. 3B), the mitochondrial network fragmentation, the Δψm dissipation (Fig. 4B) and the appearance of apoptotic nuclei (Fig. 5). Unexpectedly, we could not detect any significant differences in these parameters comparing WT and Q297V Opa-1 transfected cells. However, results obtained in Opa-1 over-expressing cells suggest that this protein would contribute to enhance Opa-1 complexes stabilization in Mn-exposed cells preventing cristae associated cytochrome c release and the subsequent events. Similar results were reported by employing Opa-1 over-expressed-MEF cells treated with different intrinsic apoptotic stimuli (1 mM H_2_O_2_, 2 µM etoposide, 2 µM staurosporine and pcDNA3.1-tBid) [51]. Likewise, it has been proven that an acute down-regulation of parkin in human SH-SY5Y cells severely affects mitochondrial morphology and ATP production and both could be rescued by over-expressing Opa-1 or a dominant negative mutant of Drp-1 [52].

As mentioned above, Opa-1 over-expression prevents the Δψm dissipation (Fig. 4B). Previous studies have demonstrated that Opa-1 isoforms containing the exon 4 not only modulate the fusion activity but also maintain the Δψm [53]. In our experiment, the isoforms of over-expressed Opa-1 hold the exon 4. In addition, it is important to stand out that the fusion process allows the recovery of mitochondria which have lost the Δψm.

Both the identification of the molecular mechanisms responsible for the mitochondrial remodeling processes during apoptosis and the determination of the proper time when fission occurs in relation to MOMP are still subjects of debate [40]. In this work, we prompted us to determine whether Δψm loss was associated to changes in mitochondrial dynamics. The pre-incubation of C6 cells with CsA prevented the increase in Mn-induced mitochondrial fission (Fig. 6C). This result suggests that MPTP opening and consequent mitochondrial membrane depolarization are events upstream to the occurrence of exacerbated fragmentation. Similar results were obtained in rat primary astrocytes [54]. In addition, we also found that CsA inhibits Drp-1 translocation to mitochondria (Fig. 6B) in accordance with the diminished fission observed in Fig. 6C. Therefore CsA mediated protection could work at two levels: decreasing Δψm dissipation through MPTP inhibition and diminishing fission reducing Drp-1 translocation.

The Mn-induced Opa-1 cleavage could also be prevented by CsA (Fig. 6A) in accordance with previous results [55] demonstrating that Δψm loss and Opa-1 cleavage induced by Ca^2+^ in HeLa cells may be prevented by CsA. Hence, these findings contribute to the elucidation of temporal events taking place during apoptosis and together with those of our group [19] suggest that MOMP precedes mitochondrial fission and probably cristae remodeling. Anyhow, much is left to understand about the temporal sequence of events governing these processes which appear to vary depending on cell type and its microenvironment [57].

As we mentioned above, Drp-1 plays a critical role in fission events. This protein normally resides in the cytoplasm but also accumulates at foci in the OMM which represent future fission sites. Hence, in response to a fission signal (e.g. apoptosis) Drp-1 translocates to mitochondria, forms spirals around the organelle and promotes the constriction that culminates in mitochondrial scission [14], [56]. In our model, we determined by western blot analysis that Mn dramatically increases Drp-1 expression levels in total lysates (Fig. 1A). Furthermore, Drp-1 signal was increased in the enriched mitochondria fraction in parallel with a corresponding decrease in the cytosol (Fig. 1B). These results, strengthened by immunocytochemical analysis (Fig. 2A) supported the Mn-induced mitochondrial fragmentation observed in Fig. 4.

The Drp-1 pro-apoptotic role was first studied in COS-7 cells treated with staurosporine to induce the intrinsic pathway [58]. This study demonstrated that over-expression of a dominant-negative (Drp-1 K38A) avoids excessive fragmentation, Δψm loss and the release of cytochrome c. Subsequently, this role was confirmed by other research groups using over-expression or silencing strategies [40], [59]–[61]. In the present work, we determine the role of Drp-1 in Mn-induced apoptosis in C6 cells by using a novel reagent (Mdivi-1) [62] and a Drp-1 silencer.

Mdivi-1 inhibits Drp-1 thus attenuating the early stages of fission, which involve the self-assembly of Drp-1 molecules. Specifically, Mdivi-1 binds selectively to a pool of Drp-1 not assembled in the cytoplasm and promotes the accumulation of a conformation unable to polymerize. Since Drp-1 self-assembly is considered a critical step for its function, Mdivi-1 was postulated as a specific GTPase activity inhibitor and finally an effective blocker of Drp-1 induced-mitochondrial fission. Considering that Mdivi-1 is also able to inhibit apoptosis, it has been proposed as a therapeutic drug based on studies conducted in models of stroke, myocardial infarction and neurodegenerative diseases [62]. Our results demonstrated that 0.001 µM Mdivi-1 exhibited a protective effect on cell death (Fig. 7A), partially preserving the integrity of Δψm and mitochondrial morphology (Fig. 7B). It also prevents the appearance of apoptotic nuclei (Fig. 7C). Therefore, our data points to Drp-1 as a player in Mn-induced apoptosis in C6 cells.

Notoriously, Mdivi-1 concentration analyzed in our studies was considerably lower than the usually one used in COS and HeLa cells [63]. Using an apoptotic model of human dopaminergic neurons it has been reported that 10 µM Mdivi-1 was the optimal concentration for maintaining ATP levels, Δψm integrity and normal mitochondrial morphology [64]. Therefore, we consider that the optimal concentration to be used may be dependent on the cellular type and/or the death stimuli.

Previous studies have demonstrated that Mdivi-1 exerts similar preventive actions to those carried out by Drp-1 siRNA and dominant-negative GTPase domain mutant, Drp-1 K38A [63]. That is the case in our model: Drp-1 silencing prevents a Mn-induced decrease in cell viability as well as the appearance of apoptotic nuclei and also preserves mitochondrial morphology and Δψm to a similar degree as Mdivi-1 (Fig. 8).

Chronic exposure to Mn leads to its accumulation mainly in the striatum and globus pallidus [4]. To the best of our knowledge, there are numerous *in vitro* studies demonstrating the involvement of apoptotic events in Mn neurotoxicity while the only *in vivo* study using striatal injections belongs to Massieu’s group [65]. These authors demonstrated the induction of cell death in striatum and globus pallidus and the activation of both caspase-3 and calpain employing intra-striatal injections of Mn. Our results showed that after 7 days of Mn-treatment, striatum undergoes evident damage judging for a moderate decrease in cellular mass. In addition, the appearance of pyknotic nuclei in the treated-animals tissue could be indicative of apoptotic cell death (Fig. 9). Finally, we evaluate the effect of Mn-striatal administration on mitochondria-shaping proteins expression after 7 days Mn-treatment (Fig. 10). Results showed a marked decrease in Opa-1 immunostaining in Mn-treated tissue in parallel with an unexpected decrease in Drp-1 immunolabeling. Similar results were reported in brain tissues from patients suffering AD showing a decrease in the Opa-1, Mfn-1, Mfn-2 and Drp-1 expression levels [66]. These authors proposed that a fusion and fission proteins altered expression pattern may contribute to mitochondrial dysfunction and neurodegeneration in AD.

Taken together our findings supported by different experimental approaches, demonstrate for the first time that a de-regulation in Opa-1 and Drp-1 expression levels is implicated in Mn toxicity in glial cells. Our *in vivo* study which included Mn administration directly in the rat striatum area seems to support the *in vitro* conclusions. A qualitatively analysis showed a preserved striatum structure 7 days after a single Mn injection, suggesting that the dose used is not a priori linked with a toxic effect at histological level. In addition we show that Mn treatment induces striatum damage as well as changes in Opa-1 and Drp-1 levels. The identification of mitochondrial-shaping proteins as well as the elucidation of mechanisms in which they are involved may contribute to the design of novel molecular intervention targets in Manganism and possibly PD.
